# Resection of an esophageal schwannoma with thoracoscopic surgery: a case report

**DOI:** 10.1186/s40792-016-0256-0

**Published:** 2016-11-07

**Authors:** Takayoshi Watanabe, Tatsuya Miyazaki, Hideyuki Saito, Tomonori Yoshida, Yuji Kumakura, Hiroaki Honjyo, Takehiko Yokobori, Makoto Sakai, Makoto Sohda, Hiroyuki Kuwano

**Affiliations:** 1 Department of General Surgical Science, Gunma University Graduate School of Medicine, 3-39-22 Showa-machi, Maebashi, Gunma 371-8511 Japan; 2 Department of Molecular Pharmacology and Oncology, Gunma University Graduate School of Medicine, 3-39-22 Showa-machi, Maebashi, Gunma 371-8511 Japan

**Keywords:** Esophageal schwannoma, EUS-FNA, FDG-PET CT, S-100, Thoracoscopic surgery

## Abstract

**Background:**

Esophageal schwannomas are rare primary submucosal esophageal tumors. We herein report a case of an esophageal schwannoma that was difficult to diagnose.

**Case presentation:**

A 39-year-old woman presented with chief complaints of difficulty swallowing and epigastric pain. Enhanced computed tomography of her chest revealed a tumor mass at the upper thoracic esophagus with internal heterogeneity. 18-Fluorodeoxyglucose positron emission tomography/computed tomography showed a hypermetabolic appearance matching the tumor mass; the accumulation had a maximum standardized uptake value of 5.5. We performed endoscopic ultrasound-guided fine-needle aspiration biopsy under general anesthesia, but the small specimens obtained prevented a definitive diagnosis. Thoracoscopic esophagectomy was performed due to the large size of the tumor, suspicion of its malignant potential, and the patient’s symptoms. Histopathological examination revealed spindle-shaped cells in a fasciculated and disarrayed architecture in the proper muscle layer. Immunohistochemical studies showed S100 protein positivity and the absence of CD34 and c-kit. We diagnosed the tumor as a benign schwannoma.

**Conclusions:**

We herein report a relatively rare case of schwannoma of the esophagus that was diagnosed with difficulty.

## Background

The incidence of benign primary tumors of the esophagus is low. The most common histological type is leiomyoma; the incidence of schwannoma among benign tumors is low [1–5]. Esophageal schwannomas are difficult to definitively diagnose by biopsy because they are located in the submucosal tissue.

18-Fluorodeoxyglucose positron emission tomography (FDG-PET) has been used to detect metabolism of glucose by tumors. High FDG uptake is generally found in malignant tumors; it is also found in inflammation. Although they are benign tumors, schwannomas reportedly show a hypermetabolic appearance on FDG-PET [6–8]. We previously reported the utility of FDG-PET for diagnosis of schwannoma of the stomach [9].

In general, surgical resection of esophageal submucosal tumors is dependent upon the tumor size, malignant potential, and patient’s symptomatic state. Although the final diagnosis must be obtained surgically, as in the present case, minimally invasive thoracoscopic surgery is considered to be an appropriate technique for esophageal submucosal tumors.

## Case presentation

**Fig. 1:**
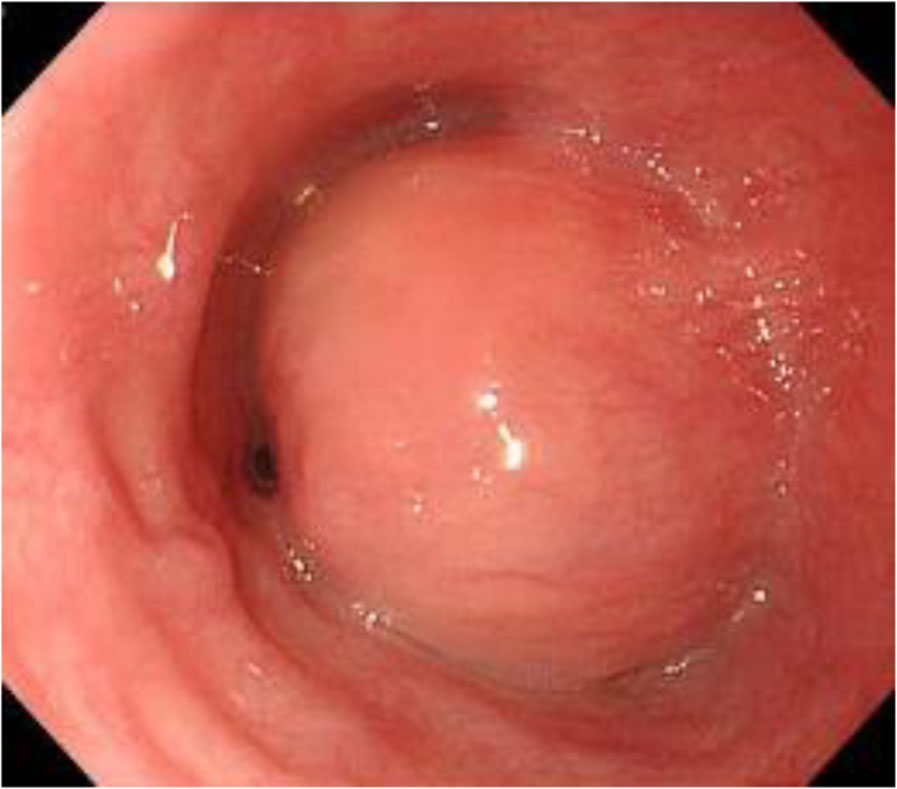
Endoscopic findings. This endoscopic photograph demonstrates the submucosal tumor of the esophagus. It occupied the most of the esophageal lumen

A 39-year-old woman presented for evaluation of difficulty swallowing and epigastric pain. Her medical and familial histories were unremarkable. Upper gastrointestinal endoscopy showed a smooth elevated lesion located 19 to 24 cm from the incisor teeth (Fig. 1). Endoscopic ultrasonography showed a large tumor mass of low echogenicity in the esophageal wall. The tumor mass originated in the muscle layer, and we performed a boring biopsy of the tumor mass. However, we were unable to obtain a tissue specimen from the tumor.

**Fig. 2:**
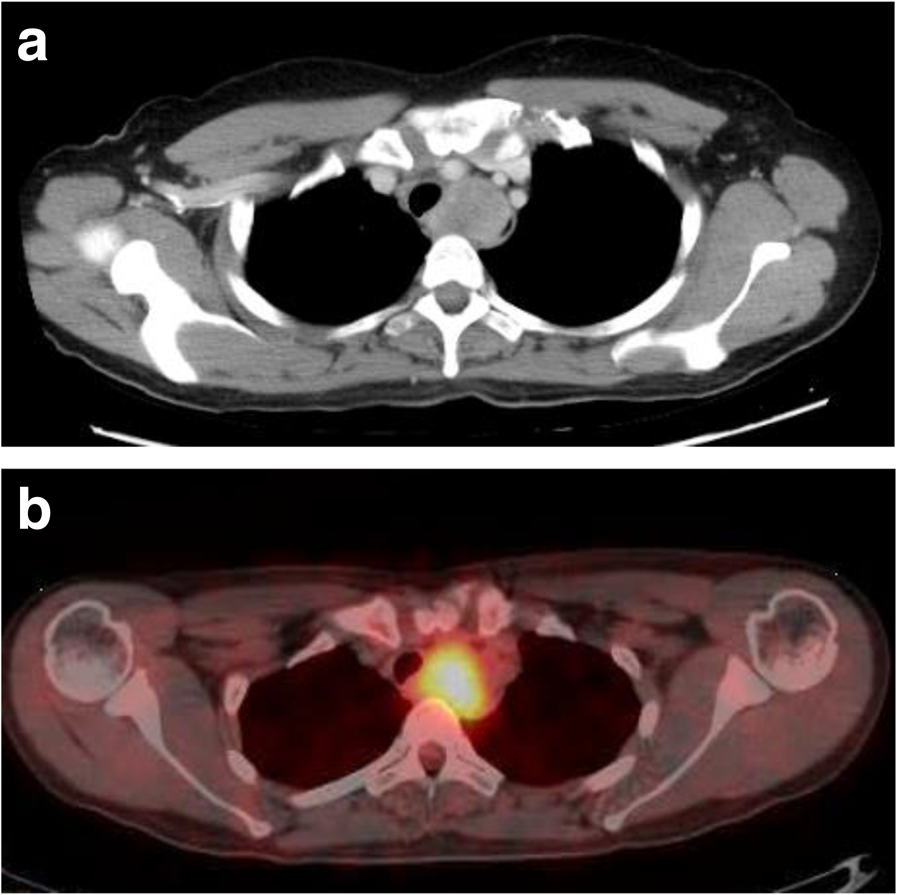
CT and FDG-PET CT findings. **a** Computed tomography revealed a large isodense mass of the esophageal wall in the upper mediastinal space. **b** Accumulation of fluorodeoxyglucose is demonstrated in the upper thoracic esophagus

A chest enhanced computed tomography (CT) scan revealed a tumor mass (39 × 28 × 56 mm) at the upper thoracic esophagus with internal heterogeneity. No metastasis to the lymph nodes or other organs was seen on the CT scan (Fig. 2a). FDG-PET CT showed a hypermetabolic appearance matching the tumor mass. The maximum standardized uptake value (max SUV) was 5.5 (Fig. 2b).

Magnetic resonance imaging of the chest revealed that the boundary of the tumor was clear and smooth. The mass exhibited isointensity or low intensity compared with the muscles on T1-weighted images and high intensity on T2-weighted images. There was no invasion into the surrounding tissue.

We performed endoscopic ultrasound-guided fine-needle aspiration biopsy (EUS-FNAB) with the patient under general anesthesia. Because the specimen was small, we were unable to establish a definitive diagnosis. We suspected an esophageal schwannoma, leiomyoma, or gastrointestinal stromal tumor (GIST).

The patient underwent surgery because of her difficulty swallowing and our suspicion of malignant potential. We performed thoracoscopic surgery with the patient under general anesthesia and single-lung ventilation.

First, we attempted enucleation of the tumor. However, the mass was larger than 5 cm, and enucleation was difficult without creating an extensive defect of the esophageal wall. Therefore, subtotal esophagectomy was performed with thoracoscopic assistance.

**Fig. 3:**
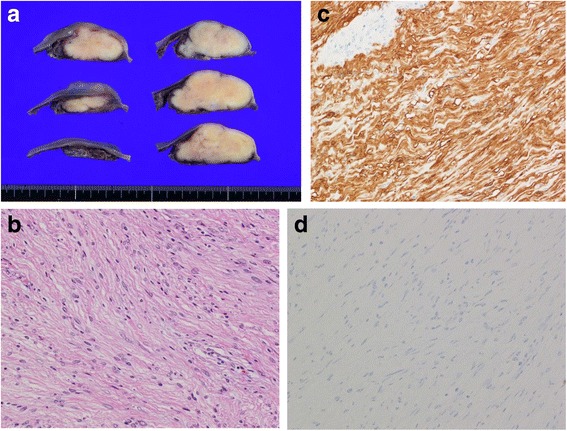
Representative photomicrographs of tissue sections. **a** Massive white tumor mass in the submucosal layer. **b** High-power photomicrograph of the tumor shows spindle-shaped cells in a fasciculated and disarrayed architecture (hematoxylin–eosin, ×400). **c** S-100 was detected in the cytoplasm of the tumor cells (×100). **d** C-kit was not detected in the tumor cells (×100).

The resected specimen measured 55 × 45 × 24 mm. The cut surface was solid milky white (Fig. 3a). Histopathological examination revealed spindle-shaped cells in a fasciculated and disarrayed architecture in the proper muscle layer. Immunohistochemical studies showed S100 protein expression and the absence of CD34 and c-kit protein expression. Nuclear division was inconspicuous, and the MIB-1 labeling index was <10 %. Finally, we diagnosed the tumor as a benign schwannoma. The patient’s postoperative course was uneventful, and she had no evidence of recurrence at the time of this writing.

## Conclusions

Benign primary tumors of the esophagus account for less than 1 % of all esophageal tumors [1]. The most common benign submucosal esophageal tumor is leiomyoma. According to previous reports, leiomyoma accounts for more than half of all benign esophageal tumors [1, 2]. Benign submucosal esophageal tumors also include lipomas, granular cell tumors, and schwannomas. A GIST is an esophageal submucosal tumor with malignant potential.

A few cases of primary esophageal schwannomas have been reported [3, 4]. Gastrointestinal schwannomas account for 0.4 to 1.0 % of all submucosal tumors of the gastrointestinal tract. Most occur in the stomach; the development of a schwannoma in the esophagus is uncommon [4].

The definitive diagnosis of an esophageal submucosal tumor should be made before tumor excision to avoid unnecessary surgery. However, the preoperative pathological diagnosis of a submucosal tumor is unclear in some cases. As in the present case, an adequate specimen is difficult to obtain during biopsy of a submucosal tumor, even if a boring biopsy is performed. This is because the surface of the submucosal tumor is often covered with normal epithelium. EUS-FNAB is now commonly performed to diagnose submucosal tumors. This technique obtains more adequate specimens than does biopsy with upper gastrointestinal endoscopy. Rong et al. [5] reported that the diagnostic accuracy of EUS-FNAB for submucosal tumors was 85.2 % [5]. However, as in our case, EUS-FNAB does not always allow for a definitive pathological diagnosis. We are unsure of the reason for the lack of an adequate specimen in our case. However, a puncture at an acute angle was required to reach the deep portion of the esophageal wall. EUS-FNAB might have been a potentially dangerous procedure in our case because the aortic artery and trachea are located behind the esophagus in the mediastinal space. This may have contributed to the difficulty obtaining the EUS-FNAB specimen in our case. We proposed that the patient undergo EUS-FNAB a third time, but she refused.

Because the preoperative pathological diagnosis was not clear in our case, we performed subtotal thoracoscopic esophagectomy to both treat the patient’s symptoms and possibly diagnose a malignancy. Even if the tumor mass had been a malignancy such as a GIST, the operation would have been curative because we performed subtotal esophagectomy.

Although the final diagnosis in this case was a benign schwannoma, FDG-PET showed a hypermetabolic appearance, suggesting malignant potential. Schwannomas are benign tumors, but a hypermetabolic appearance on FDG-PET has been reported. Schwannomas originate from nerve cells that express glucose transporter type 3 [7], and FDG uptake is considered to be increased for this reason. Beaulieu et al. [7] reported that there was no correlation between FDG uptake and the proliferation rate (Ki-67 index). The authors stated that even if the max SUV is >6.0, a benign schwannoma cannot be excluded. Our case does not conflict with their report in that our benign schwannoma showed a max SUV of 5.5 and MIB-1 labeling index of 10 %. Therefore, FDG-PET could not reveal whether the hypermetabolic appearance indicated a benign or malignant tumor. Otherwise, the findings can be meaningful for a diagnosis of schwannoma.

There is no consensus regarding the surgical treatment of esophageal submucosal tumors. Although the stomach and esophagus differ, the clinical practice guidelines for GIST in Japan declare that surgery is indicated for gastric submucosal tumors larger than 5.0 cm [10]. In our department, we first perform boring biopsy or EUS-FNAB to obtain a histopathological diagnosis of the submucosal tumor. If the submucosal tumor is malignant, such as a GIST, we usually perform a surgical operation. When the histopathological diagnosis is a benign lesion or is unclear, as in the present case, we perform one of the several approaches. If the tumor is small or asymptomatic, we usually perform simple follow-up of the patient. When the tumor is large, increasing in size, or symptomatic, we consider invasive treatment with curative intent.

We usually first try endoscopic treatment for small tumors originating in the submucosal layer. We then attempt enucleation by a thoracoscopic approach because it is less invasive. If the defect of the esophageal wall is expected to be large upon intraoperative examination or the mass has malignant potential, we perform an extended operation based on the individual patient. In this case, we considered open subtotal esophagectomy when the tumor was diagnosed as malignancy or it was difficult to resect it safely and radically.

Thoracoscopic surgery is becoming common [8]. In our department, we believe that thoracoscopic surgery is a good technique for the treatment of benign tumors because it is less invasive and clinically appropriate, especially for benign esophageal tumors. Compared with conventional operations, thoracoscopic esophagectomy for esophageal submucosal tumors is reportedly associated with fewer postoperative complications such as pneumonia and allows for earlier ambulation [11–13]. We selected the thoracoscopic approach to improve our patient’s quality of life and performed subtotal esophagectomy with curative intent.

In conclusion, we have reported a relatively rare case of an esophageal schwannoma. The diagnosis was difficult because we could not obtain an adequately sized specimen by boring biopsy and EUS-FNAB. The thoracoscopic approach is a good treatment option for large submucosal tumors of the esophagus.

## Abbreviations

CT: Computed tomography
EUS-FNAB: Endoscopic ultrasound-guided fine-needle aspiration biopsy
FDG-PET: 18-Fluorodeoxyglucose positron emission tomography
GIST: Gastrointestinal stromal tumor
max SUV: Maximum standardized uptake value
